# Political Context and Political Participation Across the Lifespan in Africa

**DOI:** 10.1093/geroni/igab046.149

**Published:** 2021-12-17

**Authors:** Eugene Dim, Markus Schafer

**Affiliations:** University of Toronto, Toronto, Ontario, Canada

## Abstract

Gerontologists have long documented how age is associated with political participation. However, few studies have considered how macrocontextual factors shape participation across the life span. Moreover, very few studies have dealt with political engagement and aging in emerging democracies, including those in Africa. This study addresses those gaps, integrating the most recent three waves of Afrobarometer survey data (2011–2018) with country-level data from the freedom house (i.e. freedom index). Findings reveal that, at the individual level, an age gap widens for engagement in protests and shrinks for electoral and non-electoral political participation. When the political context is considered, however, we find that political freedom softens the drop-off of protest behavior at later ages. For electoral and non-electoral political participation, we find that freer countries lessen the expected growth in engagement across the life span. The study implies that political oppression shapes the links between age and political behaviour, but the processes seem different depending on whether they are engaging in risky (where the age gap widens) or non-risky (where the age gap shrinks) political forms of engagement.

